# How Dutch initiatives to early discharge COVID-19 patients were organised during the pandemic: a scoping review

**DOI:** 10.1136/bmjopen-2024-097839

**Published:** 2025-08-26

**Authors:** Rick T van Uum, Martine Breteler, Hans Hardeman, Alma C van de Pol, Karin Smit, Josi A Boeijen, Abeer Ahmad, Fifianne Rigter, Roderick P Venekamp, Frans H Rutten, Dorien LM Zwart

**Affiliations:** 1 Department of General Practice & Nursing Science, Julius Center for Health Sciences and Primary Care, Utrecht, The Netherlands; 2 Department of Internal Medicine and Dermatology, UMC Utrecht, Utrecht, The Netherlands; 3 Department of Anesthesiology, UMC Utrecht, Utrecht, Netherlands; 4 Department of Pulmonology, St Antonius Ziekenhuis, Nieuwegein, The Netherlands

**Keywords:** COVID-19, Hospital to Home Transition, INFECTIOUS DISEASES, Digital Technology, Primary Health Care

## Abstract

**ABSTRACT:**

**Introduction:**

The steep rise in hospital admissions during the COVID-19 pandemic put a strain on available resources and provision of adequate healthcare globally. Translocation of care to the home setting through early discharge with remote monitoring has the potential to relieve pressure on the hospital care system, reduce costs and could simultaneously benefit patients’ well-being.

**Objective:**

Provide an overview of similarities, differences and outcomes of early discharge initiatives for COVID-19 patients in the Netherlands to foster future development of safe and effective ‘hospital at home interventions’.

**Design:**

Grey and academic literature searches from 1 March 2020 to 1 December 2024 to identify initiatives that discharged COVID-19 patients early through a remote monitoring intervention.

**Setting:**

The Netherlands.

**Participants and interventions:**

Hospitals that initiated an early discharge programme for COVID-19 patients.

**Primary and secondary outcome measures:**

We descriptively compared the available protocols to identify similarities and differences in the execution of the early discharge process. Next, we compared clinical outcomes as reported in available publications and retrieved from post-publication follow-up data collection.

**Results:**

We retrieved 15 protocols (55.6%) and six publications (5 retrospective cohorts, 1 randomised controlled trial) of 27 early discharge initiatives. Two initiatives provided follow-up data. Overall, initiatives were fairly similar, however, with differences in (i) patient selection, (ii) the responsible physician, (iii) the duration and parameters of monitoring and (iv) use of a dedicated monitoring centre. Readmission rates were low, and deaths did not occur except for one initiative (n=8, 3.8%). Patient satisfaction assessed by questionnaires was very high.

**Conclusion:**

This study provides insights into the design and potential of early discharge initiatives with remote monitoring launched in the Netherlands during the COVID-19 pandemic. Preliminary data suggest relatively low readmission and mortality rates, and good patient acceptance, but future research should provide definitive conclusions.

**Strengths and limitations of this study**

- To our knowledge, the first study of its kind to provide a comprehensive overview of different early discharge initiatives for COVID-19 patients.
- The outcomes of this study facilitate future development of such initiatives, potentially extending to home management of other infectious diseases.
- This study includes previously unpublished, real-world data, thereby allowing more in-depth investigation of early discharge initiatives.
- This study was limited to Dutch initiatives, which has implications for its generalisability.

## Introduction

 The steep rise in hospital admissions during the COVID-19 pandemic put a strain on available resources and provision of adequate healthcare globally.^1^ During the peaks of the various COVID-19 waves, intensive care wards reached their maximum capacity and usual out-patient and chronic care were halted; elective surgery and management of non-life-threatening conditions were postponed. In the wake of the pandemic, healthcare personnel shortages, especially nursing staff, were an imminent matter of concern.

Hence, this situation created urgency for alternative management options to provide healthcare for COVID-19 patients, such as early discharge and acute home-based management initiatives, reducing the demand for hospital beds and personnel. A substantial number of patients with COVID-19 admitted to the hospital could possibly be discharged early with safe remote management at home until full recovery.^2^,^4^ Translocation of care to the home setting has the potential to relieve pressure on the hospital care system, reduce costs, and if well-organised, could simultaneously benefit the well-being of patients through fewer nosocomial infections, delirium or cognitive deterioration, and anxiety/distress.^5^ Moreover, relatives are more optimally involved in the recovery process, thereby partially reducing the need for (nursing) staff involvement.

Remote monitoring is a key component of these home monitoring initiatives but has yet only been tested in patients with chronic progressive conditions with exacerbations, for example, heart failure and chronic obstructive pulmonary disease (COPD).^6^,^8^ Pulse oximetry has a central role in remote monitoring and has been shown to be an important discharge indicator in other hospital-at-home initiatives for respiratory tract infections (among others for COVID-19). Hypoxaemia measured with pulse oximetry is an easy-to-use key indicator of clinical deterioration and need for advanced care.^9^,^12^

In this scoping review, we provide an overview of similarities, differences and outcomes of early discharge initiatives for COVID-19 patients in the Netherlands to foster future development of safe and effective ‘hospital at home interventions’ with monitoring of at least oxygen saturation. For example, for patients with severe lower tract infections such as community-acquired pneumonia and severe influenza infection. We intend to contribute to the growing body of knowledge on designing remote care initiatives for acutely ill patients, thereby facilitating future development and research efforts.

## Methods

### Search strategy and inclusion criteria

We aimed to identify all Dutch early discharge initiatives for COVID-19 patients. To this end, we explored both grey and academic literature searches. First, to obtain an overview of potential initiatives, we performed a search with Google and in mainstream Dutch medical journals that the vast majority of Dutch medical doctors receive and that also have specific news sections (Medisch Contact, Nederlands Tijdschrift voor Geneeskunde), websites of Dutch hospitals, the online general practitioner (GP) Forum HAWeb and the Dutch General Practice Research Consortium.^13^ Our search of medical journals was exhaustive; we screened Google until 30 consecutive results did not yield any further new initiatives.

Next, to identify initiatives that published patient-related outcome data, we searched PubMed, Embase, Cochrane Library and clinicaltrials.gov for publications from 1 March 2020 to 1 December 2024. We used database-specific syntaxes of keywords relevant to ‘COVID-19’ and ‘remote monitoring’ or ‘home monitoring’ (see online supplemental table 1 for full descriptions of keywords) in all searches.

Initiatives were included under the following conditions: (i) patients admitted to hospital for COVID-19, (ii) availability of an early discharge protocol, and (iii) continuation of oxygen treatment at home in the majority of patients (hence reflecting patients who would normally have stayed in the hospital).

### Data collection and analysis

We extracted relevant information from the available protocols and publications. Where possible, information gaps were supplemented by contacting clinicians involved in the development of local initiatives. We tried to collect data from both published and unpublished programmes, to prevent publication bias. However, it is important to notice that most initiatives were never intended to be research projects and thus did not collect outcome data (with the exception of a few).

We constructed five themes, based on a thoroughly developed and currently running pilot study aimed at evaluating the feasibility and safety of a complex acute home management intervention:

- Eligibility of patients.
- Organisation of home treatment.
- Organisation of remote monitoring.
- Organisation of nursing care at home.
- Devices used.

For each of these categories, we descriptively compared the available protocols to identify similarities and differences in the execution of the early discharge process. Next, we compared available publications for clinical outcomes. To enrich the available data, local centres were invited to supplement their published data with anonymised data from patients that participated in the initiative after the original publication, according to a standardised case report form (see online supplemental table 2).

All data were extracted independently by two reviewers (FR and RvU) and analysed descriptively.

## Results

### Search results

We identified 27 early discharge initiatives, of which six were published.^14^,^19^ The University Medical Center (UMC) Utrecht and St Antonius hospital Nieuwegein provided additional data on request. Most publications were retrospective cohorts, with the exception of one randomised controlled trial including 62 participants: the UMC Utrecht Early@Home trial.^17^

### Protocol availability

We were able to retrieve protocols from 15 (55.6%) of the 27 early discharge initiatives (see online supplemental table 3). The main reason for failure of retrieval lay with the hospitals failing to respond despite multiple attempts by the researchers.

### Eligibility of patients

Eligibility criteria have been summarised in online supplemental table 4. In all initiatives, early discharge was offered to (i) admitted, (ii) PCR-confirmed COVID-19 patients who were (iii) clinically stable (for at least 24–48 hours or with an improving trend, assessed by treating physician) with (iv) persistent oxygen needs because of oxygen saturation (SpO2) in rest of 92%–94% reached with maximum O2 supplementation of 3–4 liter per minute (L/min)). Patients with severe comorbidities (eg, pre-existing severe lung disease) were considered not eligible for early discharge in most initiatives.

Parameters used for judging clinical stability varied but mostly consisted of a combination of breathing frequency of <20 per minute in rest, haemodynamically stable and a pulse rate of <100 beats per minute in rest. The majority of centres (11 out of 15) required that patients were able to perform activities of daily living independently and required that the patient or a close relative had decent fluency in Dutch and had adequate skills in operating a telephone app. Some also required that the patient confirmed not to smoke (at least for the duration of home treatment with oxygen supplementation).

### Organization of home treatment

All centres offered guidance for continuation of medical therapy, such as oral dexamethasone (maximum 10 days), thrombosis prophylaxis and antibiotics when indicated.

In 9 out of 15 centres, the responsibility for patients’ medical care was transferred to the GP on discharge from the hospital (*Amsterdam, Assen, Den Bosch, Den Haag, Eindhoven, Heerlen, Rotterdam-2, Tilburg, Twente*). On discharge, the treating hospital physician called the GP for a formal hand-over and asked whether the GP agreed with early discharge of that specific patient. The GP would then receive a letter to notify him/her about the patient’s personal management plan and the last vital parameters measured in the hospital. In case the GP declined, the patient stayed in the hospital.

In the remaining six centres, the treating hospital physician (pulmonologist or internist) remained responsible until the end of the COVID-19 management episode. A letter was sent to inform the GP about the patient’s inclusion in the early discharge home management programme. At the end of the COVID-19 care, a discharge letter was sent to the GP, transferring medical care back to the GP.

### Organization of remote monitoring

Patients were asked to record signs, symptoms and measurements in the Luscii app (Luscii Healthtech BV, Amsterdam, the Netherlands) three times daily (Rotterdam-2: one time, Zwolle: two times). Measurements included (i) oxygen saturation and (ii) pulse (all centres), (iii) temperature, (iv) breathing frequency, (v) general well-being score, (vi) cough score and/or (vii) shortness of breath score in some. According to the protocols, in most centres, there were three reasons for contact with the patient: (1) routine daily contact moment, (2) if certain measurements were below or above predefined threshold values, or (3) on patient request. See online supplemental table 4 for a full display of parameter thresholds for contact with patients.

Eight hospitals worked with a dedicated monitoring centre, one through an external organisation (Chipmunk International BV), which was informed by patient’s signs and symptoms via the Luscii app. Monitoring centres were available 7 days a week during working hours or until 10 PM and were staffed by trained medical students, nurses or physicians under supervision of attending physicians (either pulmonologist or internist).

In the remaining seven initiatives, monitoring was conducted via the GP or an out-of-hours (OOH) GP clinic. In these initiatives, patients were asked to record signs, symptoms and measurements in a paper diary, and provide them to the GP once she/he called.

During out-of-hours times, patients were referred to the OOH-GP clinic except for three centres (*Nijmegen, Utrecht, Zwolle*) where the on-call hospital specialist could be called directly by the patient.

Intervention decisions were mainly based on levels of oxygen saturation in all centres, complemented by a variety of other parameters (see again online supplemental table 4 for the predefined thresholds used in the various initiatives). Maximum O2 supplementation at home ranged from 3 to 5 L/min. In case of an oxygen saturation <90%–93% in rest (two repeated measurements with 30–60 min interval), an increase of O2 supplementation with 1 L/min was advised. O2 supplementation was decreased with 1 L/min if oxygen saturation was 94% or higher in most centres, or at the discretion of the GP (Assen, Den Bosch). Remote monitoring was terminated if patient’s oxygen saturation remained clinically stable without oxygen for 24–72 hours.

### Organization of nursing care at home

Across all initiatives, patients did not routinely receive nursing care at home, only if deemed necessary by hospital physician in charge (as per usual care).

### Devices used

The Luscii app is a widely used, Conformité Européenne (CE)- and Medical Device regulation (MDR)-approved medical app in the Netherlands. It hosts a variety of remote disease management programmes developed by clinicians. The app allows for tailor-made protocols and questionnaires, as well as recording of parameters. A pulse oximeter for use at home was provided in 13 out of 15 centres: *Heerlen* and *Nijmegen* provided the pulse oximeter via an external party; Chipmunk Health BV and Vivisol (oxygen supplier), respectively. Most centres (10 out of 15) did not specify the type of pulse oximeter provided. The remaining used the CE marked HUM Aerocheck (*Assen*), Westfalen HbO Smart (*Dordrecht*), Medisana (*Den Bosch*) and iHealth Air (*Zwolle and Utrecht*). The latter one is also adequately validated and Food and Drug Administration approved. Patients were asked to use their own thermometer.

### Published data

#### Study populations

Five retrospective cohorts (*Amsterdam, Dordrecht, Nieuwegein 2020, Nieuwegein 2021, Rotterdam*)^14^,^1618 19^ and one randomised controlled trial (RCT) (*Utrecht*)^17^ were published. Patient numbers ranged from 49 to 320 patients. One cohort, *Nieuwegein* 2021^15^ comprised all patients of another, previous cohort, *Nieuwegein 2020*.^14^ Consequently, four unique retrospective cohorts were available. Two hospitals (*Nieuwegein, Utrecht*) provided additional complete follow-up data on all patients managed in their early discharge initiative from inception up until December 2021 (*Nieuwegein*) or December 2022 (*Utrecht*).

#### Patient outcomes

Patient outcomes have been summarised in online supplemental table 5 and table 5 (continued). The mean age ranged from 55 to 60 years. Few patients were smokers in these initiatives (<5%). The number of patients admitted to intensive care unit prior to early discharge differed substantially across initiatives, ranging from 2.0% to 21.2%. Almost all patients were dismissed from the hospital while on oxygen therapy, except for the Nieuwegein study (60% dismissed on oxygen).

The hospital length-of-stay prior to early discharge was very short in *Rotterdam* compared with other initiatives: 1.7 days vs 8.0 to 10.6 days, respectively. The mean number of days patients received oxygen at home ranged from 3.4 (SD 7.5) to 7.7 days (SD 7.1). Avoided days in hospital were reduced with an average of 4.1 to 12.6 days. Total duration of home monitoring was similar across the initiatives and exceeded the days on oxygen treatment at home suggesting that patients were monitored longer at home than they would have been if they had stayed in the hospital.

6.5% to 18% of the patients visited the ED for a reassessment during the monitoring period, of which approximately half were readmitted (3.7 to 9.1%). In nearly all cases, readmission was warranted because of respiratory compromise. Reasons for respiratory compromise were superinfection (Aspergillus, bacterium), acute heart failure, episodic cough with desaturation, COVID-19 disease progression and pulmonary embolism.

In all but one of the initiatives, none of the patients died. In *Dordrecht,* 8 (3.8%) deaths were reported, of which six were attributed to COVID-19.

Patient satisfaction was investigated in four initiatives. In all of these initiatives, satisfaction was high, ranging from 86%–94% in *Dordrecht* to 93% in *Nieuwegein 2021* (score 0 (very low satisfaction) to 100% (maximal satisfaction)). Patient satisfaction score was 8 in *Amsterdam* and 9 in *Dordrecht* (in a score ranging from 0 to 10, 10 being maximal satisfaction) and 5 to 6 in *Rotterdam-1. Rotterdam-1* (score ranging from 0 to 6, 6 being maximal satisfaction). Aspects that patients reported as positive: (i) ease of use and display of parameters in the app, (ii) being home among their beloved ones earlier than usual and (iii) the personal contact with the nurse in the monitoring centre. *Amsterdam* explored why four patients and two GPs who reported a score lower than 5 (out of a score 0 to 10) and reported that this was caused by insufficient communication on discharge from the hospital.

Additional follow-up data from *Nieuwegein* and *Utrecht* provided a more comprehensive view as it also included patient data from later waves of the COVID-19 pandemic. These data reflected a shifting epidemiology of admitted COVID-19 patients; fewer patients receiving dexamethasone and more patients suffering from comorbidities, most notably bacterial (super-)infection in around half of patients. The number of avoided days in hospital was smaller (5 to 7.8 days) compared with earlier initiatives (12.6 days). Comparable to the prior published data, patients in *Nieuwegein* and *Utrecht* received in later COVID-19 waves oxygen at home on average 5.6 and 7.7 days, respectively, vs 11.6 and 10.9 days of home monitoring.

## Discussion

Overall, Dutch early hospital discharge initiatives for COVID-19 patients shared similarities but contained significant differences that hamper direct comparison of patient outcomes, efficiency of monitoring programmes and the effect on healthcare resource use; that is, (i) patient selection, (ii) the responsible physician, (iii) the duration of, and parameters subject to monitoring and (iv) use of a dedicated monitoring centre.

### Patient selection

The patient population in the different initiatives was heterogeneous. Some initiatives include low-risk COVID-19 patients while others specifically aimed at including high-risk patients. One initiative also included patients who did not need oxygen treatment at home.

Smokers were often excluded (due to safety reasons with supplemental oxygen) as were patients with insufficient digital or Dutch language proficiency, or those who lived without a caregiver to assist them. Irrespective of heterogeneity in patients included in the different initiatives, patient outcomes were rather similar. Also for early discharge initiatives with less frequent patient monitoring. This suggests that early discharge may be suitable for a large variety of COVID-19 patients.

### Responsible physician

Whether a hospital specialist or GP was responsible may potentially affect the efficiency of home management, but current data do not allow us to draw conclusions on which healthcare provider is most favourable in the setting of home management. One could argue that GPs are more used to make medical decisions and deal with more uncertainties in a resource-limited environment which may render them more suitable for ‘overlooking’ the patients who are early discharged. On the other hand, hospital specialists are more experienced in managing severely diseased patients needing hospital care, and as such, may be able to recognise deterioration earlier and thus improve survival (at the cost of higher readmission rates).

### Duration of monitoring

In the presented initiatives, monitoring duration was not only dependent on saturation level but also on the physician in charge. Results showed that in most patients, monitoring at home was continued while no longer dependent on oxygen for 24–72 hours which might imply that hospital physicians feel uncertainty when to discontinue home monitoring safely, resulting in less efficient healthcare resource use. This uncertainty might also pertain to patients to discontinue the monitoring. Although this specific aspect has not been assessed in this study, patient satisfaction was reported high and the personal contact with the nurse in the monitoring centre was considered important for the satisfaction. All-in-all, the extended monitoring with resulting high patient satisfaction may outweigh resource use, especially when monitoring duration might be reduced, but this has yet to be assessed.

### Monitoring center

Remote monitoring was organised in different ways, either through dedicated monitoring centres or more incidentally by the GP, with the use of an app or paper diaries, using oxygen saturation as the primary monitored variable but with many different additional parameters monitored, and with different frequencies of contact moments. Although most protocols offered daily contact with a monitoring centre/physician, in practice, contact frequency was decreased during later COVID-19 waves, likely based on increased experience in recognising which patients needed to be contacted during which period of the disease trajectory. Entry of data through a special app and through a dedicated monitoring evidently offers a lot of advantage to the healthcare provider: data are more real-time, can be presented graphically and automatic signalling of abnormal values can be implemented. However, patient preferences on paper vs app diaries and contacting their trusted GP rather than a more anonymous monitoring centre may very well be relevant factors to consider these options.

### Patient outcomes

The available five retrospective cohorts and one RCT that published patient outcomes showed similar and favourable results in terms of patient satisfaction and safety.^14^,^19^ All early discharge initiatives led to a considerable number of avoided days in the hospital at acceptable readmission rates (3.7 to 9.1%). Deaths did not occur in remotely managed patients except for the initiative of a single hospital.

All published data were collected during the first COVID-19 waves, thus prior to vaccination, when disease course of COVID-19 was in general more severe than in the post-vaccination era. Nevertheless, follow-up cohort data extending to 2023 revealed a similar perspective on patient outcomes.

Patient satisfaction assessed by questionnaires was in general very high. This has not been assessed in the follow-up cohort (ie, during later phases of the COVID-19 pandemic). However, other studies reported high patient acceptance of early discharge initiatives in other diseases.^20 21^

### Comparison with literature

Our study adds to the slim body of evidence that currently exists on remote home monitoring initiatives for acute treatment of infectious diseases such as COVID-19. Vindrola-Padros *et al*^22^ reviewed 27 available studies on remote monitoring and early discharge strategies, including *Nieuwegein-2*. They found similar results for home monitoring duration, emergency room visits, readmission and mortality, although with large variability and reporting bias, hindering the authors from drawing definite conclusions. Another systematic review yielding 28 studies on the efficacy of remote vital signs monitoring in detecting clinical deterioration of patients in the hospital at home or postacute setting did not find a significant difference in readmissions, but mortality reduced significantly.^23^ However, this was not studied in a COVID-19 population and only two studies addressed the hospital at home setting. Furthermore, there was high heterogeneity in study populations, interventions and outcomes measured. Consequently, comparison with our study remains difficult.

When comparing our study results to patients that have been admitted to the hospital alone, readmission rates and risk of death (after discharge) were lower.^24^ In a hospital-managed cohort, the readmission and death rate at 30 days were 16.0% and 10.9%, respectively. This compared with readmission rates of 3.7% to 9.1% in our studies, and mortality after discharge of less than 1%.

Home measurement of oxygen saturation levels is paramount to all these protocols, as it is proven to timely identify potentially worsening COVID-19 patients before admission^11^ and after discharge.^25 26^

### Strengths & limitations

- To our knowledge, this is the first study of its kind to provide a comprehensive overview of early discharge initiatives in patients with COVID-19 with remote home monitoring strategies within a single healthcare system.
- Completeness of data was maximised by contacting all initiatives.
- Not all initiatives could be included because some hospitals refused to provide data.
- Data pooling was not a valid option due to heterogeneity in study population and design.

Our results seem representative for the Dutch population but may be extrapolated to other healthcare systems with a similar organisational structure of primary care.

### Recommendations for practice and research

We suggest multiple key points to consider when designing early discharge initiatives in the future. First, patient selection should be well-defined and the benefits of using a digital app to monitor measurements should be weighed against the benefit of (or in addition to) phone calls. Second, responsibility of care should be well-defined and communicated among all healthcare professionals and patients involved. Third, criteria for termination of monitoring should be standardised and directed towards more efficient care. Of note, oxygen saturation levels have a central role in monitoring but have practical limitations^27^ and only validated and CE-certified metres should be used to prevent inadequate treatment decisions based on inaccurate readings.^28^ Fourth, local practices vary from region to region and therefore, it is advised to design such an initiative in collaboration with all relevant stakeholders, that is, hospital physicians, GPs, patients and home nursing organisations, preferably context-specific per region. Ideally, healthcare systems operate with one ‘master protocol’ which allows for regional variation where needed.

Overall, one should take into account that home monitoring initiatives may not result in an alleviation of healthcare use but rather to deviation (healthcare offered by different healthcare providers) or even an increase in healthcare use.^29^

In addition to the contents of an early discharge initiative, it is paramount that this is designed prior to a time-pressured environment such as a pandemic. For an early discharge initiative to function optimally, the design of a protocol should be part of an epidemic preparedness strategy rather than an ad-hoc plan during a pandemic. This is to ensure that the protocol is not only focused on coping with resource scarcity but also incorporates patients’ and healthcare providers’ preferences, organisational structures (eg, monitoring centres), data collection methods (app vs paper) and monitoring protocols that are assessed for effectiveness and safety.

Future research should support this by studying the effectiveness and safety of early discharge; preferably with a sufficiently powered randomised controlled trial. Paramount to a safe and effective early discharge initiative will be to select the right patients, for example, through validated algorithms.^30^

Furthermore, it may be of interest to explore how the current findings extrapolate to different patient categories, such as pneumonia and influenza, who may benefit from the knowledge gained through these COVID-19 home monitoring initiatives.

## Conclusions

This study provides new insights on the design and potential of early discharge initiatives in the management of COVID-19 patients. Theoretically, this might be extended to acute respiratory tract infection hospital admissions (eg, severe influenza infection, community-acquired pneumonia early discharge initiatives) and could provide potential relief of pressure on hospital bed occupancy. However, more research is definitively needed to provide solid conclusions on effectiveness and safety of such initiatives.

## Supplementary material

10.1136/bmjopen-2024-097839 online supplemental file 1

## Data Availability

Data will be made available upon reasonable request to the principal investigator.
