# Supplementary material for: How Dutch initiatives to early discharge COVID-19 patients were organised during the pandemic: a scoping review
Source: BMJ Open. 2025 Aug 26;15(8):e097839. doi: 10.1136/bmjopen-2024-097839 (PMC12382530; doi:10.1136/bmjopen-2024-097839)
Supplement: online supplemental file 1 [file bmjopen-15-8-s001.docx]

**APPENDIX TABLE 1 – SEARCH STRATEGY KEYWORDS**

| **Keywords** | **Synonyms** |
| --- | --- |
| Home monitoring | remote monitoring, at home monitoring, remote home monitoring, remote patient monitoring, home-monitoring, remote screening, Covid@home monitoring, self monitoring, self-monitoring |
| Early discharge | Early@Home, hospital at home, therapy at home, home treatment, treatment at home, early discharge, early hospital discharge |
| COVID-19 | COVID, COVID-19, Covid-19, coronavirus, SARS-CoV-2 |

**APPENDIX TABLE 2 – CASE REPORT FORM**

| **Variable** | Value | Unit |
| --- | --- | --- |
| **Baseline characteristics**  Number of patients in cohort  Male  Age (mean)  Treatment during hospital admission  -Dexamethason  -Tocilizumab/biological  Complications at/during hospital admission  *-ICU admission*  *-duration of ICU admission*  *-Pulmonary embolism*  *-Bacterial infection*  *-Heart failure*  Medical history  *-Diabetes mellitus*  *-Hypertension*  *-BMI >30*  *-COPD*  *-Cardiovascular disease*  *-Immunocompromised status*  *-Active smoker* |  | n*  n (%)  years (+-SD)  n (%)  n (%)  *n (%)*  *mean in days +-SD*  *n (%)*  *n (%)*  *n (%)*  *n (%)*  *n (%)*  *n (%)*  *n (%)*  *n (%)*  *n (%)*  *n (%)* |
| **Early discharge disease course**  Number of patients dismissed on oxygen therapy  Total treatment duration (hospital+at home)  Admission duration  Avoided days in hospital  *with O2 at home*  *without O2 at home (monitoring only)*  Telemonitoring duration  Oxygen therapy duration |  | n (%)  mean in days +-SD  mean in days +-SD  *mean in days +-SD*  *mean in days +-SD*  mean in days +-SD  mean in days +-SD |
| **Clinical endpoints**  Readmission  Indication for readmission, by diagnosis  *Respiratory deterioration/hypoxemia*  *Pulmonary embolism*  Death |  | n (%)  *n (%)*  *n (%)*  n (%) |

*n=absolute number of patients

**APPENDIX TABLE 3 – OVERVIEW OF (NON-)RETRIEVABLE PROTOCOLS**

| Available protocols  *City* | *Hospital* | Non-retrieve protocols  *City* | *Hospital* |
| --- | --- | --- | --- |
| *Amsterdam* | Cooperation of AmsterdamUMC, OLVG, BovenIJ | **Alkmaar** | Noordwest Hospital |
| *Assen* | cooperation of  Wilhelmina Hospital Assen,  Treant Care group, Emmen/Hoogeveen/Meppel,  GP care Drenthe | **Amersfoort** | Meander Medical Centre |
| *Den Bosch* | Jeroen Bosch Hospital | **Arnhem** | Rijnstate Hospital |
| *Den Haag* | Haags Medical Center and  Haga Hospital | **Breda** | Amphia Hospital |
| *Dordrecht* | Albert Schweitzer Hospital | **Ede** | Gelderse Vallei Hospital |
| *Eindhoven* | Cooperation of  Catharina Hospital Eindhoven, Maxima Medical Center Veldhoven, St. Anna Hospital Geldrop | **Gouda** | Groene Hart Hospital |
| *Heerlen* | Cooperation of  Zuyderland Medical Center Heerlen, Sittard-Geleen and Brunssum,  GPs Oostelijk Zuid-Limburg,  GPs Westelijke Mijnstreek | **Haarlem** | Spaarne Gasthuis |
| *Nieuwegein* | St. Antonius Hospital | **Harderwijk/**  **Lelystad** | St. Jansdal |
| *Nijmegen* | Canisius-Wilhelmina Hospital | **Maastricht** | Maastricht University Medical Center (MUMC+) |
| *Rotterdam-1* | Maasstad Hospital | **Venlo** | VieCuri Medical Center |
| *Rotterdam-2* | cooperation of  Maasstad Hospital,  St. Franciscus Gasthuis & Vlietland, Erasmus Medical Center,  Ikazia Hospital,  IJsselland Hospital | **Weert** | St. Jansgasthuis |
| *Tilburg* | Elisabeth-Tweesteden Hospital Tilburg and Waalwijk | **Zaandam** | Zaans Medical Center |
| *Twente* | Medisch Spectrum Twente |  |  |
| *Utrecht* | UMC Utrecht |  |  |
| *Zwolle* | Isala Hospital |  |  |

**APPENDIX TABLE 4 – ELIGIBILITY CRITERIA FOR EARLY DISCHARGE INITIATIVES OF COVID-19 PATIENTS IN THE NETHERLANDS**

| **Eligibility criteria** | **Early discharge initiatives** | | | | | | | | | | | | | | |
| --- | --- | --- | --- | --- | --- | --- | --- | --- | --- | --- | --- | --- | --- | --- | --- |
|  | *Amsterdam* | *Assen* | *Den Bosch* | *Den Haag* | *Dordrecht* | *Eindhoven* | *Heerlen* | *Nieuwegein* | *Nijmegen* | *Rotterdam-1* | *Rotterdam-2* | *Tilburg* | *Twente* | *Utrecht* | *Zwolle* |
| COVID-19 diagnosis | PCR-confirmed | | | | | | | | | | | | | | |
| Age in years | ≥18 | | | | | | | | | | | | | | |
| Clinically stable or recovering^1^ | Yes, A | No | No | Yes, A | Yes, A+B | No | Yes, A | Yes | Yes, A | No | Yes, A^3^ | Yes, A^3^ | Yes, A^3^ |  | Yes, A |
| Saturation  (SpO2, in %) | >93 | >92 | >93 | >93 | ≥94^2^ | ≥93 | >93 |  | >94 | ≥94 |  | >93 | >93 |  | ≥94^2^ |
| Maximum O2  in liters per minute | 3 | 3 | 4 | 5 | 3 | 4 | 3^4^ | 3 | 3 | 2 | 2 | 4 | 3^4^ | 3^4^ |  |
| Additional requirements on comorbidities (exclusion) | Pre-existent lung disease with oxygen need |  | Comorbidities that require prolonged admission | |  | Comorbidities that require prolonged admission |  |  |  | stable diabetes mellitus |  |  |  | *i*v medication |  |
| ADL independent | Yes^5^ | No | No | Yes | Yes | Yes | Yes^5^ | No | Yes^5^ | No | Yes | Yes^5^ | Yes^5^ | Yes | Yes |
| Caregiver at home strictly required | Yes^6^ | Yes | Yes | Yes | Yes | Yes | Yes^6^ | No | No | No | No | Yes^6^ | Yes^6^ | Yes | Yes |
| Sufficient mastery of Dutch language | No | Yes | No | No | Yes | Yes | No | No | Yes | Yes | Yes | No | No | Yes | No |
| Digital proficiency required and/or facilities available at home | No | Yes | Yes | No | Yes | No | No | No | Yes | Yes | No | No | No | Yes | Yes |
| GP approval required | Yes | No | Yes | Yes | Yes | Yes | No | No | No | No | Yes | No | No | No | No |
| Non-smoker | No | No | Yes | No | Yes | No | Yes | Yes | Yes | No | No | No | No | No | No |
| Other |  |  | No smoking |  | Admitted for >24 hrs | Admitted to COVID-ward | Admitted to COVID-ward |  |  |  |  |  |  | COVID is the primary indication for admission | >7 days after initial COVID-symptoms |

*^1^Defined as A) stable blood pressure, breathing frequency <20 per minute and known, stable blood glucose level or B) no signs of type 2 respiratory failure (pCO2 >6,65 kPa, decreased pO2 and pH <7,35)*

*^2^ Or alternatively: saturation ≥92% without oxygen if acceptable clinical parameters*

*^3^ for at least 48 hours*

*^4^ Oxygen delivery has been stable for at least 24 hours (Utrecht) or 48 hours (Heerlen, Twente)*

*^5^ Patient is either capable of performing daily living activities independently or, if not, care is delivered by caregiver or nurse*

*^6^ In case patient is not fully capable of performing daily living activities independently, a home care nurse will provided care otherwise delivered by caregiver*

**APPENDIX TABLE 4 (I/II) – PARAMETER THRESHOLDS FOR CONTACT WITH PATIENTS – EARLY DISCHARGE**

| **Hospital** | **Amsterdam** | **Assen** | **Den Bosch** | **Den Haag** | **Dordrecht** | **Eindhoven** | **Heerlen** |
| --- | --- | --- | --- | --- | --- | --- | --- |
| **Parameter** |  |  |  |  |  |  |  |
| **Saturation in %** | <92 | ≤90 (red)  ≤92 (orange)  -2 | ≤94 (two times) OR  ≤89 | ≤92 | ≤91 | <92 | <92 |
| **Increased oxygen need when in rest** |  |  | YES | YES |  | YES | YES |
| **Breathing frequency**  **per minute** | >11 | ≥12 | >11 | >14 | ≥10 | >11 |  |
| **Temperature in °C** | new fever | ≥38.5 | new fever | new fever | ≥38.5 | new fever | new fever |
| **Heart rate in beats per minute** |  | >100 |  |  |  |  |  |
| **Cough score**  **(0-10 points)** |  |  |  |  |  |  |  |
| **Shortness of breath score**  **(0-10 points)** |  |  |  |  |  |  |  |
| **Shortness of breath** | ↑ |  | ↑ | ↑ |  | ↑ | ↑ |
| **General wellbeing** |  | if negative | ↓ | ↓ |  | ↓ | ↓ |
| **Value not measured** |  | any |  |  |  |  |  |
| **Blood pressure** |  |  |  |  |  |  |  |
| **Elevated glucose despite treatment** | YES |  | YES | YES |  | YES | YES |
| **New thoracic pain** | YES |  | YES | YES |  | YES | YES |

**APPENDIX TABLE 4 (II/II) – PARAMETER THRESHOLDS EARLY DISCHARGE**

| **Hospital** | **Nieuwegein** | **Nijmegen** | **Rotterdam1** | **Rotterdam2** | **Tilburg** | **Twente** | **Utrecht** | **Zwolle** |
| --- | --- | --- | --- | --- | --- | --- | --- | --- |
| **Parameter** |  |  |  |  |  |  |  |  |
| **Saturation**  **(%)** | ≤94 (two times)  ≤89 | ≤93 (two times)  ≤88 | <94  -2 | <92 | <92 | <92% | individual threshold  -2 | <94  -2 |
| **Increased oxygen need when in rest** |  |  |  | YES | YES |  |  |  |
| **Breathing frequency**  **per minute** |  |  |  | >12 | >11 | >11 | ↑ in rest |  |
| **Temperature in °C** | new fever |  | >37.0 | new fever | new fever | new fever | ≥38.0 | >37.9 |
| **Heart rate in beats per minute** |  |  |  |  |  |  |  |  |
| **Cough score**  **(0-10 points)** |  |  | >5  +2 |  |  |  | ≥6  ≥+2 | >3  +2 |
| **Shortness of breath score**  **(0-10 points)** | ↑ |  |  |  |  |  | ≥6  ≥+2 | >5  +2 |
| **Shortness of breath** | ↑ |  |  | ↑ | ↑ | ↑ |  |  |
| **General wellbeing** | ↓ |  |  | ↓ | ↓ | ↓ | ↓ | ↓ |
| **Value not measured** |  |  |  |  |  |  |  |  |
| **Blood pressure** |  |  |  |  |  |  |  |  |
| **Elevated glucose despite treatment** |  |  |  |  | YES | YES |  |  |
| **New thoracic pain** | YES |  |  | YES | YES | YES |  |  |
| **Other** |  |  |  |  | Delirium |  |  |  |

**APPENDIX TABLE 5 – PUBLISHED BASELINE DATA OF EARLY DISCHARGED COVID-19 PATIENTS IN THE NETHERLANDS**

| **Variable** | **Unit** | **Hospital** |  |  |  |  |  |  | |  |  |
| --- | --- | --- | --- | --- | --- | --- | --- | --- | --- | --- | --- |
|  |  | **Amsterdam** | **Dordrecht** | **Nieuwegein** | | | **Rotterdam-1** | **Utrecht** | | | |
|  |  | *AUMC, OLVG, Bovenij* | *Albert Schweitzer* | *Antonius* | | | *Maasstad* | *UMCU (Early@Home)* | | | |
|  |  | 2021^16^ | 2022^19^ | JAMIA **2020**^14^ | ERJ **2021**^15^ | 2023  *Follow-up†* | 2021^18^ | 2021^17^ | | | 2023  *follow-up†* |
|  |  |  |  |  | *Also comprises 2020 cohort* |  |  | *Intervention* | *Control* | |  |
| **Baseline characteristics** |  |  |  |  |  |  |  |  |  | |  |
| Number of patients in cohort | n | 113 | 213 | 33 | 320 | 339 | 49 | 31 | 31 | | 75 |
| Age | mean years +- SD | 58* | 59.9 ± 11.4 | 57 +- 12 | 56 +- 12 | 56.3 +- 12.3 | 56 +- 12.3 | 55.1 +- 7.5 | 55.4 +- 13.2 | | 57.7 +- 9.8 |
| Male | n (%) |  | 131 (61.5) | 22 (66.7) | 206 (64.4) | 220 (64.9) | 25 (51.0) | 17 (54.8) | 18 (58.1) | | 51 (68) |
|  |  |  |  |  |  |  |  |  |  | |  |
| Treatment during admission |  |  |  |  |  |  |  |  |  | |  |
| *Dexamethasone* | *n (%)* | *113 (100)* | *197 (92.5)* | *33 (100)* | *320 (100)* | *275 (81)* | *49 (100)* | *31 (100)* | *31 (100)* | | *73 (97.3)* |
|  |  |  |  |  |  |  |  |  |  | |  |
| Complications at/during admission | n (%) |  |  |  |  |  |  |  |  | |  |
| *ICU admission* | n (%) |  | 12 (5.6) | 7 (21.2) | 54 (16.8) | 57 (16.8) | 1 (2.0) | 4 (12.9) | 3 (9.7) | | 11 (14.7) |
| *Duration of ICU admission* | mean days +- SD |  | 4 (2-7)* | 8 (5-14)* | 9 +- 8.5 | 9.3 +- 8.5 |  |  |  | | 8 +- 5.9 |
| *Pulmonary embolism* | n (%) |  |  |  |  | 20 (5.9) |  | 2 (6.5) | 3 (9.7) | | 5 (6.7) |
| *Bacterial infection* | n (%) |  |  |  |  | 175 (51.6) |  | 2 (6.5) | 3 (9.7) | | 40 (53.3) |
| *Acute heart failure* | n (%) |  |  |  |  | 5 (1.5) |  |  |  | | 0 (0) |
| *Other* |  |  |  |  |  |  |  | 3 (9.7) | 1 (3.2) | |  |
|  |  |  |  |  |  |  |  |  |  | |  |
| Medical history | n (%) |  |  |  |  |  |  |  |  | |  |
| *DM* |  |  | 18 (8.5) |  | 39 (12.2) | 44 (13.0) |  |  |  | | 15 (20) |
| *Hypertension* |  |  |  |  | 89 (27.8) | 98 (28.9) |  | 6 (19.4) | 5 (16.1) | | 19 (25.3) |
| *BMI >30* |  |  | 35 (42.7) |  | 100 (31.3) | 113 (33.3) |  |  |  | | 27 (36) |
| *COPD* |  |  | 12 (5.6) |  | 90 (28.1) | 96 (28.3) |  |  |  | | 5 (6.7) |
| *chronic heart disease* |  |  | 69 (32.4) |  | 54 (16.9) | 60 (17.7) |  | 2 (6.5) | 3 (9.7) | | 14 (18.7) |
| *immunocompromised* |  |  | 2 (0.9) |  | 10 (3.1) | 11 (3.2) |  |  |  | | 13 (17.3) |
| *smoker* |  |  |  |  |  | 17 (5.0) |  | 1 (3.2) | 0 (0) | | 2 (2.7)** |

^†^ *additional follow-up data delivered by hospitals of all patients managed in the early discharge programme from inception up and until December 2022. These follow-up data also comprises patient data from prior publications*

** median with interquartile range (IQR) instead of mean*

*** missing n=44*

**APPENDIX TABLE 5 – PUBLISHED OUTCOME DATA OF EARLY DISCHARGED COVID-19 PATIENTS IN THE NETHERLANDS**

| **Variable** | **Unit** | **Hospital** |  |  |  |  |  |  | | |  | |
| --- | --- | --- | --- | --- | --- | --- | --- | --- | --- | --- | --- | --- |
|  |  | **Amsterdam** | **Dordrecht** | **Nieuwegein** | | | **Rotterdam-1** | **Utrecht** | | | | |
|  |  | *AUMC, OLVG, Bovenij* | *Albert Schweitzer* | *Antonius* | | | *Maasstad* | *UMCU (Early@Home)* | | | | |
|  |  | 2021^16^ | 2022^19^ | JAMIA **2020**^14^ | ERJ **2021**^15^ | 2022  *Follow-up†* | 2021^18^ | 2021^17^ | | | | 2023  *Follow-up†* |
|  |  |  |  |  | *Also comprises 2020 cohort* |  | | *Intervention* | *Control* | *Difference*  *mean (95%CI)* | | *Intervention* |
| **Baseline characteristics** |  |  |  |  |  |  |  |  |  |  | |  |
| Number of patients in cohort | n | 113 | 213 | 33 | 320 | 339 | 49 | 31 | 31 |  | | 75 |
| Age | mean years +- SD | 58* | 59.9 ± 11.4 | 57 +- 12 | 56 +- 12 | 56.3 +- 12.3 | 56 +- 12.3 | 55.1 +- 7.5 | 55.4 +- 13.2 |  | | 57.7 +- 9.8 |
| Male | n (%) | N/A | 131 (61.5) | 22 (66.7) | 206 (64.4) | 220 (64.9) | 25 (51.0) | 17 (54.8) | 18 (58.1) |  | | 51 (68) |
| **Early discharge disease course** |  |  |  |  |  |  |  |  |  |  | |  |
| Number of patients dismissed  while on oxygen | n (%) | 113 (100) | 213 (100) | 20 (60.7) | 196 (61.3) | 213 (62.8) | 49 (100) | 31 (100) | 5 (16.1) |  | | 94 (98.7) |
|  |  |  |  |  |  |  |  |  |  |  | |  |
| Total treatment duration  *(admission plus oxygen at home)* | mean days +- SD |  |  | 19.5 +- 9.6 | 18 +- 9.1 | 15.5 +- 9.0 |  | 14.1 +- 7.6 | 10 +- 7.0 | 4.1 (0.5 to 7.7)  p=0.028 | | 21.1 +- 11.7 |
| Admission duration | mean days +- SD | 8* | 4 (2-7)* | 10.6 +- 7.0 | 8.6 +- 6.6 | 9.4 +- 6.5 | 1.7 (0.9-3.3) *,** | 7.3 +- 4.3 | 10 +- 7.0 | -2.7 (-5.7 to 0.0)  p=0.045 | | 10.2 +- 6.3 |
| Oxygen therapy duration | mean days +- SD | 8 (4-14)* | 10 (6-16) |  |  | 5.6 +- 5.0 | 11 (7-14) ^*^ | 6.7 +- 7.5 | 3.4 +- 7.5 | 3.3 (-0.5 to 6.8)  p=0.101 | | 7.7 +- 7.1 |
| Telemonitoring duration | mean days +- SD |  |  | 13.4 +- 4.1 | 11.7 +- 5.4 | 11.6 +- 5.3 | 12.6 |  |  |  | | 10.9 +- 7.3 |
|  |  |  |  |  |  |  |  |  |  |  | |  |
| Avoided days in hospital | mean days +- SD |  |  |  |  | 5.0 +- 3.4 | 12.6 |  |  |  |  | 7.8 +- 7.1 |
| *with O2 at home* | mean days +- SD |  |  | 6.5 | 6.4 +- 3.2 | 6.2 +- 3.1 |  |  |  |  |  | 7.8 +- 7.1 |
| *without O2 at home* | mean days +- SD |  |  | 4.1 | 5.1 +- 3.4 | 1.9 +- 1.0 |  |  |  |  |  | 0*** |
| **Clinical endpoints** |  |  |  |  |  |  |  |  |  |  | |  |
| Contact moments with monitoring center or GP, per patient | median, IQR | 6 (IQR N/A) | 11.5 (3-24) |  |  | 0 (0) | 3 (2-4) |  |  |  | | 0 (1) |
| Contact moments with GP, per cohort | n (%) |  |  |  |  | 22 (6.5) |  | 25 (80.6) | 22 (71.0) |  | | 38 (50.6) |
| ED visits |  |  | 37 (15.8) | 6 (18) | 39 (12) | 44 (13.0) |  | 1 (3.3) | 2 (6.5) |  | | 2 (2.7) |
| Readmission | n (%) | 4 (3.7) | 14 (6.5) | 3 (9.1) | 23 (7.2) | 26 (7.7) | 6 (12.2) | 2 (6.5) | 1 (3.3) | 6 (8) | | 6 (8.0) |
| Death | n (%) | 0 (0) | 8 (3.8) | 0 (0) | 0 (0) | 0 (0) | 0 (0) | 0 (0) | 1 (3.3) |  | | 1 (1.3) |

^†^ *additional follow-up data delivered by hospitals of all patients managed in the early discharge programme from inception up and until December 2022. These follow-up data also comprises patient data from prior publications*

** median with interquartile range (IQR) instead of mean*

*** hours instead of days*

**** only one single patient*
