# Supplementary material for: How Dutch initiatives to early discharge COVID-19 patients were organised during the pandemic: a scoping review
Source: BMJ Open. 2025 Aug 26;15(8):e097839. doi: 10.1136/bmjopen-2024-097839 (PMC12382530; doi:10.1136/bmjopen-2024-097839)
Supplement: Supplementary file 2 [file bmjopen-2024-097839.reviewer_comments.pdf]

PEER REVIEW HISTORY

BMJ Open publishes all reviews undertaken for accepted manuscripts. Reviewers are asked to complete a checklist review form and are provided with free text boxes to elaborate on their assessment. These free text comments are reproduced below.

ARTICLE DETAILS

Title (Provisional)

How Dutch initiatives to early discharge COVID-19 patients were organized during the pandemic: a scoping review

Authors

van Uum, Rick T.; Breteler, Martine; Hardeman, Hans; van de Pol, Alma C.; Boeijen, Josi; Ahmad, Abeer; Rigter, Fifianne; Smit, Karin; Venekamp , Roderick; Rutten, Frans H; Zwart, D.L

VERSION 1 - REVIEW

**Reviewer**	**1**
**Name**	**Jaswal, Harpreet**
**Affiliation**	**UCalgary**
**Date**	**15-Apr-2025**
**COI**	**None**

<u>Summary of research</u>

The authors of this study sought to collate and compare literature on early discharge initiatives for COVID-19 patients in the Netherlands to assist in the future development of other remote monitoring or hospital-at-home initiatives. Similarities and differences between initiatives, and relevant patient outcomes were investigated by means of a narrative review of peer-reviewed reports and gray literature. The authors found that the initiatives were relatively similar in their execution with high patient experience, while readmission and mortality remained low. The authors did an excellent job characterizing available Dutch literature and have seemed to have provided a relatively comprehensive review.

<u>Discussion of specific areas for improvement</u>

Major issues

- Introduction

- Given the specific selection of the Netherlands as the setting of interest, the authors can better highlight the need for home monitoring initiatives in this country by specifically describing the impact of the pandemic on its healthcare system, particularly during the earlier waves.
- Page 5, line 24-27; the importance of monitoring oxygen levels is mentioned in the discussion, however it may be beneficial for the authors to explicitly state the applicability and significance of this biometric measurement in relation to COVID-19 and hospital-to-home initiatives (HAH) (i.e., oxygen levels as a traditional discharge indicator for HAHs). This is particularly pertinent given the authors goal of fostering the future development of HAH initiatives with at least the monitoring of oxygen saturation. Furthermore, adding additional information here would better explain inclusion condition #3.

- Methods
  - The authors stated that they independently contacted local centres who provided anonymized patient data to enrich available date. Can the authors please state whether consultation with a REB was sought, and whether it was confirmed that ethics approval was not required to collect and use the aforementioned data?
- Results
  - The authors clearly state recommendations and interpretations throughout the discussion, however given the specificity of the study and the number of protocols included, these suggestions would be strengthened if they were supplemented by comparing to and citing other global studies. For example, the authors suggest that patient heterogeneity may make early discharge initiatives suitable for a large variety of patients. Are there other studies that have shown similar results? If so, from where? Comparing to other studies with similar health systems (e.g., Germany, Denmark) would strengthen the validity and applicability of your suggestions.

Minor issues

- Methods
  - Can the authors clarify if the same keywords listed in Appendix, Table 1 were used when non-systematically searching for relevant articles?
  - The authors stated they first Google alongside other selected Dutch resources, and then biomedical databases (i.e., PubMed, Embase). Can the authors why they sought grey literature first, and its associated benefits? Additionally, when were grey literature searches considered exhausted? For example, were searches in Google terminated when no relevant results were obtained after 50 consecutive results, and why?
  - The Medish Contact and Nederlands Tijdschrift voor Geneeskunde were listed as examples of 'mainstream Dutch medical journals'. What qualifies them as

mainstream? If specific criteria and strategies was used, this may be of use to other countries who may also consult similar grey literature.
    - Can the authors please list, describe, and cite the SQUIRE 2.0 checklist that was used in the methods section?
- Results
    - For readability, the authors may wish to organize protocol (un)availability into a table.
    - Page 8, Line 55, Page 9, Line 30: Instead of including the hyperlink, it is recommended that the authors cite the app and its relevant information instead.
    - Page 9, Line 23: Can the authors please clarify whether this finding was consistent across all included protocols?
    - Page 16, Lines 12-15: please provide a citation.
    - Page 16, section ‘patient outcomes’: given that the patient experience data was collected during the earlier phases of the pandemic, the authors would benefit from detailing how temporality may have affected or skewed the results.
- General
    - Some typos throughout the paper (e.g., page 10, line 38-39, page 13, line 4, page 15, line 41)
    - In tables 1, 2, and 3, in areas where there is missing data the authors indicating this through leaving the cell blank, ‘-‘, or N/A. For readability purposes, it is recommended to remain consistent in labelling.
    - Table 1, Page 11, instead of placing the criteria in the annotations, please list these as subcategories under ‘clinically stable or recovering’ and categorize the articles as such instead of using superscripts

---

**Reviewer**	**2**
**Name**	**Vural, Sevilay**
**Affiliation**	**UMCG, Emergency Medicine**
**Date**	**18-Apr-2025**
**COI**	**None**

Dear Authors,

This study is a valuable paper of early discharge and remote monitoring practices for patients with acute COVID-19 lung infections during the regarding pandemic. The analysis evaluates both similarities and differences among initiatives while critically assessing the safety of early discharge protocols. Surely, variations across programs in terms of patient

selection criteria, monitoring frequency, and the healthcare provider responsible for follow-up (e.g., hospital specialists vs. general practitioners) are inevitable. Also,

the data are limited to the Netherlands and include grey literature, only 55.6% of protocols were included. These are significant limitations as stated in the limitation section as well.

I found the paper very compelling. However, the discussion section is a bit dull which does not offer any comparisons and/or speculations. Surely, the paper data covers the accessible covid-19 early discharge protocols, itself. You may consider to add some additional literature papers adressing early and safe discharge on infected patients (i.e., DOI: 10.1016/j.ajem.2024.10.014) and/or remote monitoring of non-covid infections for deeper and more sofisticated discussion ground.

Despite these constraints, the study offers insightful conclusions and detailed protocol descriptions that will inform future research in similar or divergent healthcare settings. The findings provide key implication aspects, such as the role of telemonitoring and patient outcomes, contributing significantly to the literature.

In conclusion, I believe it meets the standards for publication but needs a minor revision on discussion section.

VERSION 1 - AUTHOR RESPONSE

Reviewer: 1 Ms. Harpreet Jaswal, UCalgary

Summary of research

The authors of this study sought to collate and compare literature on early discharge initiatives for COVID-19 patients in the Netherlands to assist in the future development of other remote monitoring or hospital-at-home initiatives. Similarities and differences between initiatives, and relevant patient outcomes were investigated by means of a narrative review of peer-reviewed reports and gray literature. The authors found that the initiatives were relatively similar in their execution with high patient experience, while readmission and mortality remained low. The authors did an excellent job characterizing available Dutch literature and have seemed to have provided a relatively comprehensive review.

We gratefully thanks the reviewer for the positive comments.

Discussion of specific areas for improvement

Major issues

- Introduction

o Given the specific selection of the Netherlands as the setting of interest, the authors can better highlight the need for home monitoring initiatives in this country by specifically describing the impact of the pandemic on its healthcare system, particularly during the earlier waves.

Prompted by this comment, we made the impact of the pandemic on Dutch healthcare more specific in the revised Introduction section:
During the peaks of the various COVID-19 waves, intensive care wards reached their maximum capacity and usual out-patient and chronic care were halted.

o Page 5, line 24-27; the importance of monitoring oxygen levels is mentioned in the discussion, however it may be beneficial for the authors to explicitly state the applicability and significance of this biometric measurement in relation to COVID-19 and hospital-to-home initiatives (HAH) (i.e., oxygen levels as a traditional discharge indicator for HAHs). This is particularly pertinent given the authors goal of fostering the future development of HAH initiatives with at least the monitoring of oxygen saturation. Furthermore, adding additional information here would better explain inclusion condition #3.

We thank for the reviewer's suggestion and we now explicitly stated the applicability and significance of pulse-oximetry in HAH:
Pulse-oximetry has a central role in remote monitoring and has been shown an important discharge indicator in other hospital-at-home initiatives (amongst others for COVID-19). Hypoxemia measured with pulse-oximetry is an easy to use key indicator of clinical deterioration and need for advanced care.

<u>- Methods</u>
o The authors stated that they independently contacted local centres who provided anonymized patient data to enrich available data. Can the authors please state whether consultation with a REB was sought, and whether it was confirmed that ethics approval was not required to collect and use the aforementioned data?

As per BMJ Open's requirements, we have mentioned consultation with REB in the 'Ethics approval section' rather than the Methods section. Because we used de-identified data, and patients did not undergo any intervention related to this study, informed consent or formal ethical approval was not needed according to Research Ethical Board.

<u>- Results</u>
o The authors clearly state recommendations and interpretations throughout the discussion, however given the specificity of the study and the number of protocols included, these suggestions would be strengthened if they were supplemented by comparing to and citing other global studies. For example, the authors suggest that patient heterogeneity may make early discharge initiatives suitable for a large variety of patients. Are there other studies that have shown similar results? If so, from where? Comparing to other studies with similar health systems (e.g., Germany, Denmark) would strengthen the validity and applicability of your suggestions.

We thank the reviewer and highlighted other (early discharge) initiatives in the revised Discussion.

<u>Minor issues</u>

- Methods

o Can the authors clarify if the same keywords listed in Appendix, Table 1 were used when non-systematically searching for relevant articles?

This has now been clarified:
We used database-specific syntaxes of keywords in all searches.

o The authors stated they first Google alongside other selected Dutch resources, and then biomedical databases (i.e., PubMed, Embase). Can the authors explain why they sought grey literature first, and its associated benefits? Additionally, when were grey literature searches considered exhausted? For example, were searches in Google terminated when no relevant results were obtained after 50 consecutive results, and why?

We considered grey literature important because we aimed to cover all initiatives in the Netherlands. If we had a hit on Google, we contacted the principal investigator of the initiative. We have now provided further clarification regarding the searches in the revised manuscript:
Our search of medical journals was exhaustive; we screened Google until 30 consecutive results did not yield any further new initiatives.

o The Medish Contact and Nederlands Tijdschrift voor Geneeskunde were listed as examples of 'mainstream Dutch medical journals'. What qualifies them as mainstream? If specific criteria and strategies was used, this may be of use to other countries who may also consult similar grey literature.

This we have now specified in the revised manuscript:
First, to obtain an overview of potential initiatives, we performed a search with Google, and in mainstream Dutch medical journals that the vast majority of Dutch medical doctors receive and that also have specific news sections (Medisch Contact, Nederlands Tijdschrift voor Geneeskunde).

o Can the authors please list, describe, and cite the SQUIRE 2.0 checklist that was used in the methods section?

As per the editor's request, we have now included the PRISMA-ScR checklist.

- Results

o For readability, the authors may wish to organize protocol (un)availability into a table.

Prompted by this comment, we now added Table 1 in the revised manuscript containing this information.

o Page 8, Line 55, Page 9, Line 30: Instead of including the hyperlink, it is recommended that the authors cite the app and its relevant information instead.

We removed the hyperlink and now cite the app and its relevant information:

The Luscii app is a widely used CE and MDR-approved medical app in the Netherlands that hosts a variety of remote disease management programs developed by clinicians. The app allows for tailor-made protocols and questionnaires, as well as recording of parameters.

o Page 9, Line 23: Can the authors please clarify whether this finding was consistent across all included protocols?

This has now been clarified:
Across all initiatives, patients did not routinely receive nursing care at home, only if deemed necessary by the hospital physician in charge (as per usual care).

o Page 16, Lines 12-15: please provide a citation.

We have provided the following citation:
The available three retrospective cohorts and one RCT that published patient outcomes showed similar and favorable results in terms of patient satisfaction and safety.[14,15,17]

o Page 16, section 'patient outcomes': given that the patient experience data was collected during the earlier phases of the pandemic, the authors would benefit from detailing how temporality may have affected or skewed the results.

This has now been addressed in the revised manuscript:
Patient satisfaction assessed by questionnaires was in general very high. This has not been assessed in the follow-up cohort (i.e. during later phases of the COVID pandemic). However, other studies reported high patient acceptance of early discharge initiatives in other diseases[20,21]

<u>- General</u>
o Some typos throughout the paper (e.g., page 10, line 38-39, page 13, line 4, page 15, line 41)

We thank the reviewer and have corrected these typos throughout the paper.

o In tables 1, 2, and 3, in areas where there is missing data the authors indicating this through leaving the cell blank, '-', or N/A. For readability purposes, it is recommended to remain consistent in labelling.

We have adjusted this and have chosen for '-'.

o Table 1, Page 11, instead of placing the criteria in the annotations, please list these as subcategories under 'clinically stable or recovering' and categorize the articles as such instead of using superscripts.

Since we feel that this revision would comprise readability, we refrained from adjusting Table 1 accordingly. We will leave it to the publication design team of BMJ Open whether they desire an adaptation as suggested.

Reviewer: 2 Dr. Sevilay Vural, UMCG
This study is a valuable paper of early discharge and remote monitoring practices for patients with acute COVID-19 lung infections during the regarding pandemic.
The analysis evaluates both similarities and differences among initiatives while critically assessing the safety of early discharge protocols. Surely, variations across programs in terms of patient selection criteria, monitoring frequency, and the healthcare provider responsible for follow-up (e.g., hospital specialists vs. general practitioners) are inevitable. Also,
the data are limited to the Netherlands and include grey literature, only 55.6% of protocols were included. These are significant limitations as stated in the limitation section as well.
I found the paper very compelling. However, the discussion section is a bit dull which does not offer any comparisons and/or speculations. Surely, the paper data covers the accessible covid-19 early discharge protocols, itself. You may consider to add some additional literature papers addressing early and safe discharge on infected patients (i.e., DOI: 10.1016/j.ajem.2024.10.014) and/or remote monitoring of non-covid infections for deeper and more sophisticated discussion ground.
Despite these constraints, the study offers insightful conclusions and detailed protocol descriptions that will inform future research in similar or divergent healthcare settings. The findings provide key implication aspects, such as the role of telemonitoring and patient outcomes, contributing significantly to the literature.
In conclusion, I believe it meets the standards for publication but needs a minor revision on discussion section.

We thank the reviewer for the positive remarks and suggestions.
Prompted by the comments, we have revised the strengths and limitations paragraph (as per our reply to editor's comment #3) and the Discussion section, and we added the suggested reference[30]

VERSION 2 - REVIEW

**Reviewer**	**1**
**Name**	**Jaswal, Harpreet**
**Affiliation**	**UCalgary**
**Date**	**28-Jun-2025**
**COI**	

The authors have adequately addressed my previous commentary, and I recommend this article for publication.

**Reviewer**	**2**
**Name**	**Vural, Sevilay**

Affiliation UMCG, Emergency Medicine

Date 11-Jul-2025

COI

Dear Authors,

I enjoyed reading and reviewing your article. I believe that the revisions made have resulted in a version acceptable for publication.

Best regards,
